# The Anxious Brain: The Influence of Stress on the Nervous System

**DOI:** 10.3390/brainsci14060597

**Published:** 2024-06-13

**Authors:** Drozdstoy Stoyanov

**Affiliations:** 1 Department of Psychiatry and Medical Psychology, Medical University Plovdiv, 4002 Plovdiv, Bulgaria; drozdstoy.stoyanov@mu-plovdiv.bg; 2 Research Institute, Medical University Plovdiv, 4002 Plovdiv, Bulgaria; 3 Strategic Research and Innovation Program for the Development of MU-PLOVDIV–(SRIPD-MUP), European Union–NextGenerationEU, 4002 Plovdiv, Bulgaria

Anxiety disorders, including generalized anxiety, panic disorder, and post-traumatic stress, constitute the most frequent mental disorders and occur in about 14–18% of the overall population. They can greatly affect the quality of life, especially in young adults. Thus, in this Special Issue, we aim to examine the interrelations between environmental stress factors, such as psychological and biological stressors (oxidative stress) and substance misuse, and their impact on brain structure and function. Additionally, we consider the pivotal role of shifting forms of stress in modern society due to dynamic changes in lifestyle.

It is clear that anxiety and mood disorders also share some clinical features and underlying mechanisms, notably depressive and anxiety disorders, which occur as a consequence of adaptation challenges and in response to critical stressful life events. This raises questions about endogenic conditions and “psychogenic” psychopathological reactions, according to Karl Jaspers [1]. Furthermore, distress and disability have been identified as leading causes of mental disorders on a conceptual basis [2] in addition to operational criteria, which are subject to critical considerations as regards their validity [3].

Therefore, our Special Issue is open to contributions explaining the mechanisms and stress–diathesis interactions across the entire spectrum of affective disorders and their comorbidities. The influence of stress may be seen and interpreted bi-directionally in psychosomatic dynamic relationships—from the brain to other systems and from disruptions of another system’s homeostasis to the brain. Stress in itself is a multifaceted phenomenon that is induced by both environmental and intrinsic mechanisms. The latter are assumed to be endogenic and psychogenic, and the effects of environmental and endogenic factors are mediated by neurotoxic oxidative stress and neuro-immune activation pathways [4].

The following nine manuscripts are included as part of this Special Issue:

1. Isaeva, E.R.; Ryzhova, D.M.; Stepanova, A.V.; Mitrev, I.N. Assessment of Suicide Risk in Patients with Depressive Episodes Due to Affective Disorders and Borderline Personality Disorder: A Pilot Comparative Study. *Brain Sci.* **2024**, *14*, 463. https://doi.org/10.3390/brainsci14050463.
2. Stoyanova, K.; Stoyanov, D.; Petrov, S.; Baldzhieva, A.; Bozhkova, M.; Murdzheva, M.; Kalfova, T.; Andreeva, H.; Taskov, H.; Vassilev, P.; et al. Conversion and Obsessive–Phobic Symptoms Predict IL-33 and IL-28A Levels in Individuals Diagnosed with COVID-19. *Brain Sci.* **2023**, *13*, 1271. https://doi.org/10.3390/brainsci13091271.
3. Maes, M.; Rachayon, M.; Jirakran, K.; Sodsai, P.; Sughondhabirom, A. Lower Nerve Growth Factor Levels in Major Depression and Suicidal Behaviors: Effects of Adverse Childhood Experiences and Recurrence of Illness. *Brain Sci.* **2023**, *13*, 1090. https://doi.org/10.3390/brainsci13071090.
4. Almulla, A.F.; Abdul Jaleel, A.-K.K.; Abo Algon, A.A.; Tunvirachaisakul, C.; Hassoun, H.K.; Al-Hakeim, H.K.; Maes, M. Mood Symptoms and Chronic Fatigue Syndrome Due to Relapsing-Remitting Multiple Sclerosis Are Associated with Immune Activation and Aberrations in the Erythron. *Brain Sci.* **2023**, *13*, 1073. https://doi.org/10.3390/brainsci13071073.
5. Maes, M.; Abe, Y.; Sirichokchatchawan, W.; Suwimonteerabutr, J.; Sangkomkamhangd, U.; Almulla, A.F.; Satthapisit, S. The Cytokine, Chemokine, and Growth Factor Network of Prenatal Depression. *Brain Sci.* **2023**, *13*, 727. https://doi.org/10.3390/brainsci13050727.
6. Maes, M.; Almulla, A.F. Research and Diagnostic Algorithmic Rules (RADAR) and RADAR Plots for the First Episode of Major Depressive Disorder: Effects of Childhood and Recent Adverse Experiences on Suicidal Behaviors, Neurocognition and Phenome Features. *Brain Sci.* **2023**, *13*, 714. https://doi.org/10.3390/brainsci13050714.
7. Grinevica, A.; Udre, A.; Balodis, A.; Strumfa, I. Tic Cough in an Adolescent with Organic Brain Pathology—A Case Report and Literature Review. *Brain Sci.* **2024**, *14*, 79. https://doi.org/10.3390/brainsci14010079.
8. González-Castro, T.B.; Genis-Mendoza, A.D.; López-Narváez, M.L.; Juárez-Rojop, I.E.; Ramos-Méndez, M.A.; Tovilla-Zárate, C.A.; Nicolini, H. Gene Expression Analysis in Postmortem Brains from Individuals Who Died by Suicide: A Systematic Review. *Brain Sci.* **2023**, *13*, 906. https://doi.org/10.3390/brainsci13060906.
9. Shamabadi, A.; Karimi, H.; Cattarinussi, G.; Moghaddam, H.S.; Akhondzadeh, S.; Sambataro, F.; Schiena, G.; Delvecchio, G. Neuroimaging Correlates of Treatment Response to Transcranial Magnetic Stimulation in Bipolar Depression: A Systematic Review. *Brain Sci.* **2023**, *13*, 801. https://doi.org/10.3390/brainsci13050801.

In the context of the above Introduction, one relevant set of papers in this collection [https://doi.org/10.3390/brainsci13091271; https://doi.org/10.3390/brainsci13050727] demonstrates how biological alterations in peripheral immune regulatory mechanisms are associated with mood disorders, depression, and anxiety. A number of networks and pathways are implicated, such as cytokines, chemokines, and nerve growth factor, which have an impact on conditions like conversion disorder and obsessive–phobic phenomena in patients with COVID-19, pre-natal depression, major depression, and chronic fatigue syndrome. Particularly, both remitting multiple sclerosis as an autoimmune condition and depression appear to share the mechanisms and causal pathways of immune activation.

The next area of concern in this Special Issue is suicidal behavior. Suicide and self-harm are central risks in the treatment of mental disorders and are typically investigated in terms of their complex determinants from a wide range of explanations, spanning from molecular and genetic factors to psychological risk. Genetic factors are assessed post-mortem by the expression of mRNA for genes regulating glutamate, γ-amino-butyric acid, and polyamine system brain-derived neurotrophic factor–tropomyosin-related kinase B (TrkB). The aberrations of neuro-inflammatory pathways in patients with suicidal behaviors, involving decreases in nerve growth factor in association with the re-occurrence of illness (longitudinal course of disease) and adverse childhood experiences as psychological predictors, are reported in vivo as well. Furthermore, psychosocial predictors of suicide in different populations are evaluated in terms of motivation and protective factors as concerns social orientation, demonstrativeness, self-centeredness, anxiety, and fear of death. The results are presented in several papers [https://doi.org/10.3390/brainsci14050463; https://doi.org/10.3390/brainsci13071090].

A crucial concern raised in many of the articles in this Special Issue is poor diagnostic and prognostic validity in psychiatry, which has established mono-disciplinary conventional matrices with controversial criteria and measures of obtaining diagnostic units [5]. As a result, some authors in our Special Issue argue that psychiatry as a medical discipline needs sound, nomothetic networks based on machine learning to revise current systems of classification [https://doi.org/10.3390/brainsci13050714]. Nomothetic networks form the standard operational basis for most medical disciplines, integrating knowledge and data from many disciplines. Those include but are not limited to biochemistry, pathophysiology, imaging, clinical manifestation, and public health (e.g., defining the impact of disease on the quality of life and well-being), thereby setting the stage for a data-driven approach to classification [6], prognosis, and multidisciplinary personalized case management.

Multidisciplinary team effort in psychiatry requires perspectives on all the determinants of mental health and disorders, including molecular and physiological mechanisms, as well as clinical, neurocognitive, phenomenological, and psychosocial indicators. Such measures may deliver a means of grading and classifying illness into stages, as has been adopted in other branches of medicine. This, of course, does not underestimate the critical role of supervening social and cultural influences, which also shape diagnostic frameworks.

Neuroimaging as a potential tool to define treatment targets and define the monitoring of responses is highlighted in another contribution [https://doi.org/10.3390/brainsci13050801]. Treatment response to whichever form of therapy—whether psychological, pharmacological, or via transcranial magnetic stimulation—has been a challenge in psychiatry for several decades. Current treatment strategies and guidelines are exclusively empirical and have predominantly evaluative components. Randomized controlled trials are reported using scores from rating scales, which are highly subjective, dependent on introspective and inter-subjective contexts, and confounded to a great extent by cultural, ethnic, and religious values [7]. This undermines confidence in the guidelines entailed by such trials. Therefore, the introduction of stable and data-driven therapeutic targets remains of paramount significance for this field. As an example of a response addressing these, this collection of articles is complemented by one case study describing a case of an adolescent with atrophy of the corpus callosum, who presented with a debilitating dry cough that demonstrated a somatic manifestation of organic brain syndrome.

In conclusion, the articles from this Special Issue provide insights and scope outlines that raise awareness of the explanatory mechanisms, pathways, and relevant fingerprints of disease, which converge into the impact of psychological and somatic stressors on anxiety and affective spectrum disorders.
